# Upregulated miR-10b-5p as a potential miRNA signature in amyotrophic lateral sclerosis patients

**DOI:** 10.3389/fncel.2024.1457704

**Published:** 2024-11-07

**Authors:** Banaja P. Dash, Axel Freischmidt, Anika M. Helferich, Albert C. Ludolph, Peter M Andersen, Jochen H. Weishaupt, Andreas Hermann

**Affiliations:** ^1^Translational Neurodegeneration Section “Albrecht Kossel”, Department of Neurology, University Medical Center Rostock, Rostock, Germany; ^2^Department of Neurology, Ulm University, Ulm, Germany; ^3^German Center for Neurodegenerative Diseases (DZNE), Ulm, Germany; ^4^Department of Clinical Science, Neurosciences, Umeå University, Umeå, Sweden; ^5^Division of Neurodegeneration, Department of Neurology, Mannheim Center for Translational Neurosciences, Medical Faculty Mannheim, Heidelberg University, Mannheim, Germany; ^6^Center for Transdisciplinary Neurosciences Rostock, University Medical Center Rostock, Rostock, Germany; ^7^German Center for Neurodegenerative Diseases (DZNE) Rostock/Greifswald, Rostock, Germany

**Keywords:** amyotrophic lateral sclerosis, human induced pluripotent stem cells, motor neurons, microRNA, next generation RNA sequencing, differentially expressed

## Abstract

Amyotrophic lateral sclerosis (ALS) is a fatal, adult-onset disease marked by a progressive degeneration of motor neurons (MNs) present in the spinal cord, brain stem and motor cortex. Death in most patients usually occurs within 2–4 years after symptoms onset. Despite promising progress in delineating underlying mechanisms, such as disturbed proteostasis, DNA/RNA metabolism, splicing or proper nucleocytoplasmic shuttling, there are no effective therapies for the vast majority of cases. A reason for this might be the disease heterogeneity and lack of substantial clinical and molecular biomarkers. The identification and validation of such pathophysiology driven biomarkers could be useful for early diagnosis and treatment stratification. Recent advances in next generation RNA-sequencing approaches have provided important insights to identify key changes of non-coding RNAs (ncRNAs) implicated with ALS disease. Especially, microRNAs (miRNAs) have emerged as key post-transcriptional regulators of gene expression to target several genes/pathways by degrading messenger RNAs (mRNAs) or repressing levels of gene expression. In this study, we expand our previous work to identify top-regulated differentially expressed (DE)-miRNAs by combining different normalizations to search for important and generalisable pathomechanistic dysregulations in ALS as putative novel biomarkers of the disease. For this we performed a consensus pipeline of existing datasets to investigate the transcriptomic profile (mRNAs and miRNAs) of MN cell lines from iPSC-derived *SOD1*- and *TARDBP* (TDP-43 protein)-mutant-ALS patients and healthy controls to identify potential signatures and their related pathways associated with neurodegeneration. Transcriptional profiling of miRNA–mRNA interactions from MN cell lines in ALS patients revealed differential expression of genes showed greater vulnerability to KEAP1-NRF2 stress response pathway, sharing a common molecular denominator linked to both disease conditions. We also reported that mutations in above genes led to significant upregulation of the top candidate miR-10b-5p, which we could validate in immortalized lymphoblast cell lines (LCLs) derived from sporadic and familial ALS patients and postmortem tissues of familial ALS patients. Collectively, our findings suggest that miRNA analysis simultaneously performed in various human biological samples may reveal shared miRNA profiles potentially useful as a biomarker of the disease.

## 1 Introduction

Amyotrophic lateral sclerosis (ALS) is a progressive and irreversible neurodegenerative disease characterized by the selective loss of motor neurons (MNs) in the motor cortex, brain stem and spinal cord. The majority of ALS cases are sporadic (sALS), with the remaining 5–10% of cases being familial (fALS). To date, more than 40 Mendelian inherited genes have been associated with ALS. Among those, mutations in *C9orf72*, *SOD1*, *FUS, TARDBP*, and *TBK1* are the most prevalent in European populations and have the highest penetrance (Taylor et al., 2016). These causative genes encode proteins with different functions, which are implicated with protein homeostasis, defects in stress response, aberrant RNA metabolism, and epigenetic changes including microRNAs (miRNAs) processing and function (Liu et al., 2022; Wilson et al., 2023). Despite extensive research, underlying pathomechanisms causing MN degeneration and cell death are poorly understood.

MiRNAs are small (~20–23 nucleotides in length) non-coding RNAs that negatively regulate gene expression post-transcriptionally by guiding the RNA induced silencing complex (RISC) to the 3′-untranslated region (UTR) of specific mRNAs resulting in degradation and/or translational repression of the target mRNAs (Bartel, 2018). A single miRNA can target multiple genes and a network of miRNAs may regulate the same target gene, making them important regulators of key cellular functions and homeostasis (Friedman et al., 2009). Importantly, miRNAs are well preserved and highly stable in a wide range of biological fluid/tissue/matrices, including blood plasma, serum, and cerebrospinal fluid (CSF) bound to specific proteins or as exosomal cargoes such as secreted small extracellular vehicles (EVs) (Xu et al., 2012). Despite being rapidly degraded in postmortem tissues (Sethi and Lukiw, 2009), miRNAs are resistant to RNAse degradation and extreme conditions of pH/temperature or multiple freeze–thaw cycles (Condrat et al., 2020; Grasedieck et al., 2012; Mitchell et al., 2008), thus making them useful as clinically relevant biomarkers for neurological diseases (Roy et al., 2022). Interestingly, the regulation of gene expression by miRNAs is essential for the maintenance of MNs survival and physiological functions/pathways emphasizing their importance in this highly specialized cell type (Haramati et al., 2010), which could explain, at least partially, complex neurodegenerative diseases such as ALS. Moreover, mutations in ALS-associated genes including, *TARDBP*, *FUS*, and *SOD1* activate a stress response pathway that leads to general dysregulation in miRNA expression levels, which most likely contributes to MN degeneration in ALS pathophysiology (Emde et al., 2015). In fact, in ALS patients different miRNA profiles have been identified at different stages of the disease (Gentile et al., 2022; Kmetzsch et al., 2022; Panio et al., 2022).

High-throughput (HT) transcriptomic profiling based next generation RNA-sequencing (NGS-RNA-seq) of miRNAs has become a widely used approach these days for studying post transcriptional gene regulation in ALS. With the help of NGS-RNA-seq, the functional implications of miRNAs based on induced pluripotent stem cells (iPSCs)-derived MNs have gained increasing interests. Previously, we identified dysregulated miRNAs in iPSC-derived SOD1 and TARDBP mutant MNs, and integrated miRNA dysregulation with mRNA expression data derived from the same samples (Dash et al., 2022, 2024). In this study using datasets from above mentioned published study by us, we aimed to investigate the transcriptomic profile (mRNA and miRNA sequencing data) of mutant MNs from the same ALS patients by employing different analytical methods for normalization and statistical testing. We generated a consensus pipeline to evaluate the usefulness and accuracy of different post-analytic methods commonly used for differentially expressed gene (DEG) analysis with the overall aim to find out the top hit rather than multiple numbers. We identified the most robustly dysregulated miRNAs and biological pathways enriched in the target genes shared between different forms of ALS, and validate the top candidate miRNA in different patient-derived biomaterials of sALS and fALS patients, suggesting it as a putative future biomarker.

## 2 Materials and methods

### 2.1 Ethics statement and patient characteristics

Analyses of postmortem samples from fALS patients were performed in accordance with the declaration of Helsinki. Informed written consent was provided by all participating individuals. For the postmortem autopsy material, the next of kin gave permission for material to be collected and used for medical research purposes.

Blood samples were drawn in accordance with the Declaration of Helsinki (WMA, 1964), and study protocols were approved by the national medical ethical review boards. Participants provided prior written informed consent. ALS patients were diagnosed according to the revised El Escorial criteria (Brooks et al., 2000), and genotyping was performed as described previously (Zondler et al., 2016). ALS patients were considered sporadic cases due to a negative family history over three generations and absence of mutations in known ALS genes, respectively. sALS patients were not related to any of the control individuals. Additionally, the two most frequent causes of fALS (Brown and Al-Chalabi, 2017; Hardiman et al., 2017), namely mutations in the *SOD1* and *C9ORF72* were removed by Sanger sequencing (van Es et al., 2010) or by repeat-primed PCR (DeJesus-Hernandez et al., 2011).

### 2.2 iPSC generation and differentiation into MNs

All cell lines described here have been previously published and characterized (Dash et al., 2024). Briefly, fibroblast cell lines were established from skin biopsies obtained from familial ALS patients (two of each carrying *SOD1* and *TARDBP*, respectively) which were compared to three different healthy control individuals (different families with different mutations; *SOD1* hom. D90A, *SOD1* het. R115G, *TARDBP* het. S393L and *TARDBP* het. G294V, respectively; different controls from different families, for details see also Supplementary Table S1). In case of the control lines, genetic testing was performed and they were only included if this was negative for mutations in the four most frequent ALS genes *C9ORF72*, *SOD1*, *FUS* and *TARDBP*. The reprogramming procedure to obtain iPSCs from fibroblasts and characterization of control iPSC lines was described previously (Bursch et al., 2019; Kreiter et al., 2018; Naumann et al., 2018). All procedure had been approved by the local ethics committee (EK45022009).

The generation of human neural progenitor cells (NPC) and MNs was accomplished following the protocol from Bursch et al. (2019), Naumann et al. (2018), Reinhardt et al. (2013). Importantly, the NPC culture was a resource for final MN differentiation, which was initiated by treatment with 1 μM purmorphamine (PMA) in N2B27 and supplemented with 1 μM retinoic acid (RA) on the third day. To increase the purity of MN enriched cell culture another split was performed on day 9 of the protocol. In parallel, the medium constitution was changed: Instead of PMA and RA, 10 ng/μl BDNF, 500 μM dbcAMP and 10 ng/μl GDNF was added to N2B27 ensuring neuronal maturation. All the iPSC lines we used were low passage number (less than 20) and differentiated for 14–21 days of terminal differentiation (= total DIV 30). All these cell lines have been previously characterized including the acquisition of classical spinal motor neurons markers, electrophysiological function and the sequential appearance of progressive neurodegeneration (Günther et al., 2022; Kreiter et al., 2018; Naujock et al., 2016). In summary, immunolabeling detected the presence of neuronal (TuJ1 80–90%, MAP2 80–90%) and motor neuron specific markers (SMI32 70–75%) in the cells without significant differences in neuron morphologies between wildtype controls (*n* = 3 subjects, 1 clone each) and ALS mutants (*SOD1 n* = 2 subjects, 1 clone each; *TARDBP n* = 2 subjects, 2 clones of one subject and 1 clone of second subject). There was no difference between wildtype and mutants *SOD1* (Günther et al., 2022; Naujock et al., 2016) and *TARDBP* (Kreiter et al., 2018).

### 2.3 Lymphoblastoid cell lines

Epstein–Barr virus (EBV) transformed Lymphoblastoid cell lines (LCLs) were generated from blood samples of healthy controls as well as sporadic and genetically defined ALS patients using standard procedure (Frisan et al., 2001). For this study, LCLs from 14 sALS patients, with a negative family history and no mutations in known ALS genes, 14 fALS patients (8 *SOD1*: 2 D90A, I104F, E100K, G72S, I113T, H43R, V87A; 3 *C9orf72*; 2 *FUS*: R514G, K510R; 1 *TARDBP*; N352S), and 14 healthy controls matched for age and gender were used (Supplementary Table S3).

### 2.4 Subjects and tissue

Native frozen brainstem tissue samples were derived from six fALS (3 *SOD1*: 3 D90A, G127X) and (2 *C9ORF72*) and three age- and gender-matched neurologically-healthy controls, respectively (Supplementary Table 2). All fALS patients autopsied at the clinical science department, Umeå Univeristy. We had no details on postmortem delays, but there was no significant difference in the age at death or RNA integrity number (RIN) values, a measure of tissue quality, between the groups. RIN mean values were, ALS patients = 3.46 ± 0.6754 (mean ± S.D) and healthy controls = 2.77 ± 0.6351 (mean ± S.D). *p*-value of a *t*-test comparing the RIN was 0.1711.

### 2.5 RNA isolation and RT-qPCR

RNA isolation from LCLs and brain stem tissue was carried out with the miRNeasy Mini Kit (Qiagen) according to the manufacturer’s protocol. For RT-qPCR, we used the miScript PCR System including predesigned primer assays for RNU6B and hsa-miR-10b-5p (Qiagen) according to the instructions. PCRs were run in duplicates on a CFX96 Real-Time System (Bio-Rad), and resulting Ct values of miR-10b-5p were normalized to RNU6B using 2^−ΔΔCt^ method (Livak and Schmittgen, 2001).

### 2.6 Bioinformatics analysis for total RNA and small RNA-Seq

Raw sequence FASTQ files were imported into *Partek™ Flow™* software, v11.0 for initial processing. Quality control analysis (QA/QC) were performed on all reads to assess read quality and to determine the amount of trimming required (both ends) on the positional base at which the PHRED quality score calls below 20. A minimum read length filter retaining reads ≥15 bases in length was used in this study. Trimmed reads were aligned to miRBase mature miRNAs version 22 (Kozomara and Griffiths-Jones, 2014) of the human genome assembly, hg38 (GRCh38, EnsEMBL assembly v100) using the Bowtie 2 v2.2.5 aligner (Langmead et al., 2009). A seed mismatch limit of 1 and minimum seed length of 10 were used and processed via Partek’s default parameter settings in miRNA-Seq analysis. The aligned reads were then quantified to the transcriptome through an expectation maximization (EM) method (Xing et al., 2006). Next, the raw read counts from miRNA genes were normalized and scaled to Trimmed Mean of M values (TMM) (Robinson and Oshlack, 2010), median-of-ratios (DESeq2) (Love et al., 2014) and Partek’s gene specific analysis (GSA) algorithm quantile normalization methods for comparison of the large-scale expression data across the different samples in *Partek™ Flow™* software, v11.0. Finally, normalized read counts for each miRNA were statistically modeled and differentially expressed (DE) miRNAs (DE-miRNAs) (│log_2_FC│ ≥ 1.5 or │log_2_FC│ ≤ −1.5, false discovery rate (FDR) ≤ 0.05, *p*-value ≤0.05) between healthy controls and mutant cells were identified by using three differential algorithms: the negative binomial distribution Wald test in DESeq2 (Love et al., 2014), the exact test variants in limma-voom (Ritchie et al., 2015) and the GSA approach (Wu et al., 2013) using the quantile normalization method. The intersection of the DESeq2, limma-voom and GSA results was performed by finding their common miRNAs and a Venn diagram was created to offer a graphic representation of the outcomes. For miRNA-mRNA integration analysis, the enrichment *p*-values (*p*-value ≤0.05) were calculated and adjusted by multiple test FDR correction statistical method between DE-miRNAs and DE-mRNAs using *Partek™ Genomics Suite™* software v7.0.

The total RNA sequencing datasets were obtained from our previously published study (GSE210969) (Dash et al., 2022). FASTQ files were processed and analysed based on the human reference genome [hg38//GRCh38 (obtained from EnsEMBL assembly v100)] using the STAR v2.7.8a aligner (Dobin et al., 2013). Quality control analysis (QA/QC) were performed on all files to assess read quality before and after filtering. Processed aligned reads were then quantified against the EnsEMBL v100 hg38/GRCh38 human reference genome using the Expectation Maximization (EM) algorithm (Xing et al., 2006). Normalization and differential gene expression of the raw read counts were performed using DESeq2 (v1.16.1) at the gene level (Love et al., 2014). DE-mRNAs between two groups (mutant MNs versus healthy controls) were determined based on │log_2_FC│ ≥ 1.5 and with a threshold of significance of *p*-value ≤ 0.05 and FDR ≤ 0.05. RNA-seq analysis was carried out using *Partek™ Flow™* software v11.0. All differential expression analysis results are provided in Supplementary Tables S4, S5.

### 2.7 miRNA target genes and functional enrichment analysis

We retrieved experimentally validated mRNA targets of miRNAs from miRTarBase v9.0 (Huang et al., 2022) and DIANA-TarBase v8.0 (Karagkouni et al., 2018) and integrated them with DE-mRNAs selected from the GSE210969 dataset. *Partek™ Flow™* software, v11.0. and InteractiVenn tool (Heberle et al., 2015) were used to generate the Venn diagrams for the overlapping candidate target mRNAs/genes.

To better interpret the possible pathways and target genes of the relevant miRNA-mRNA profiles of the *SOD1*- and *TARDBP* MNs, gene ontology (GO) functions and pathway [Reactome and Kyoto Encyclopedia of Genes and Genomes (KEGG)] enrichment analyses of candidate genes were carried out by utilizing the EnrichR through its web interface (Xie et al., 2021). Functional enrichment (enrichment testing across all GO functions, pathways) significance of the DEGs was analyzed by using Fisher’s exact test with adjusted *p*-values (Benjamini and Hochberg FDR ≤ 0.05) and significance was ranked by enrichment score (− log_10_ (*p*-value)). In EnrichR, we combined the signaling pathways from two different libraries, including KEGG and Reactome, to create a single analysis route. Only the important pathways for which the *p*-values ≤0.05 were evaluated and considered after deleting duplicate pathways. For functional GO annotations, we looked at the GO biological process (BP), GO molecular function (MF), and GO cellular component (CC) datasets in EnrichR and selected the most important GO terms based on set criteria. Cytoscape v3.10.1 (Shannon et al., 2003) was used to generate and visualize complex miRNA-gene regulatory network with a *p*-value ≤0.05 significance. Additional details of significantly implicated pathways and GO terms are reported in Supplementary Table S6.

### 2.8 Statistical analysis

The general pipeline for miRNA-seq analysis used in this study, including alignment, quantitation, normalization, and differential gene expression analysis as well as statistical analyses including Venn comparisons, Hierarchical clustering (with Euclidean distance measure and average linkage clustering), visualization maps and QC assessment of DE-miRNAs and DE-mRNAs were performed on the *Partek™ Flow™* software, v11.0 to aid the visualization and interpretation of the expression patterns of DE-miRNA datasets. When appropriate, *p*-values (*p*-value ≤0.05) were calculated and adjusted for multiple testing using FDR correction statistical method between DE-miRNAs and DE-mRNAs using *Partek™ Genomics Suite™* software v7.0. The outcomes were described using mean ± standard deviation (SD) and standard error of the mean (SEM) once the data were verified for the normal distribution. Unpaired, two-tailed Student’s *t*-tests were performed using GraphPad Prism v9.4.1. software to analyse experiments. *p*-values/FDRs ≤0.05 were considered statistically significant.

## 3 Results

### 3.1 miR-10b-5p is robustly upregulated in *SOD1* and *TARDBP* mutant MNs

The differentiation of iPSC-derived MNs of three different cell lines from healthy subjects and two ALS patient cell lines carrying *SOD1* (homozygous D90A mutation [one clone], heterozygous R115G mutation [one clone]) and *TARDBP* (both heterozygous S393L, G294V [two clones from patient]) mutations as well as the NGS-RNAseq had already been established previously (Dash et al., 2024). For the latter, we identified miRNAs dysregulated in *SOD1* and *TARDBP* mutant MNs based on RPKM (Reads Per Kilobase per Million mapped reads) normalization method only (Dash et al., 2024). We now hypothesized that a bioinformatics pipeline comparing three alternative methods for normalization of miRNA expression will help us to narrow down the top-regulated DE-miRNAs to the most important and generalisable pathomechanistic dysregulations in ALS. To do so, we performed a bioinformatic pipeline comparing the following three alternative methods for normalization of miRNA expression analysis, i.e., DESeq2 (median-of-ratios) (Love et al., 2014), limma-voom (TMM) (Robinson and Oshlack, 2010), and Partek’s Gene Specific Analysis (GSA) algorithm (quantile normalization) (Supplementary Figures S1) in our recently published dataset (Supplementary Table S1) (GSE210969).

**Figure 1:**
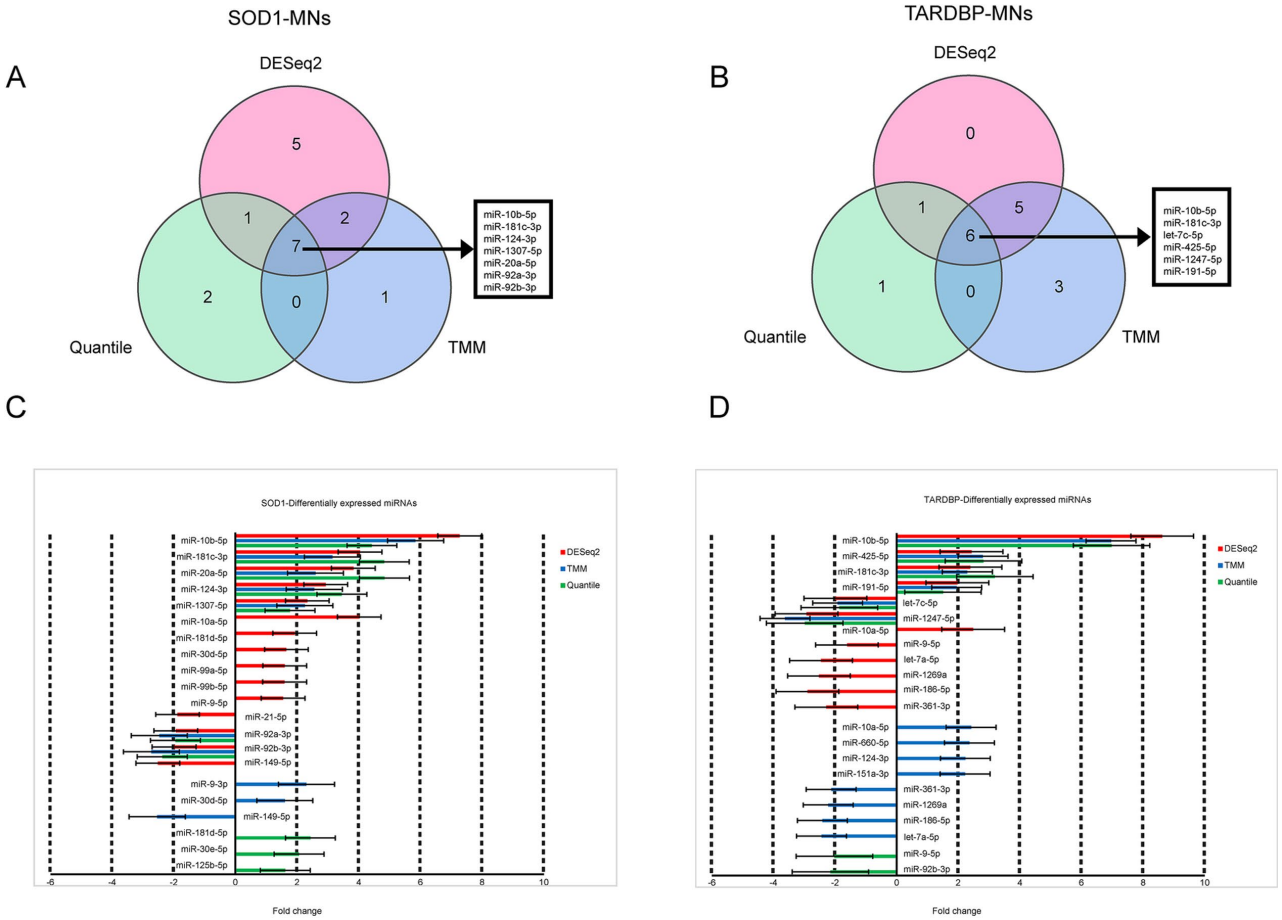
Differential expression of *SOD1* and *TARDBP* mutant MN miRNAs. (A) Venn diagrams showing DE-miRNAs identified by three normalization methods of DESeq2, TMM, and quantile at FDR ≤ 0.05, *p*-value ≤0.05 and log_2_FC ≥ 1.5 or log_2_FC ≤ −1.5 for *SOD1* MNs versus healthy controls and (B) *TARDBP* MNs versus healthy controls. (C) Differentially expressed miRNAs and the corresponding fold changes with *SOD1* MNs and (D) *TARDBP* MNs. Bars indicate mean ± S.E.M.

**Figure 2:**
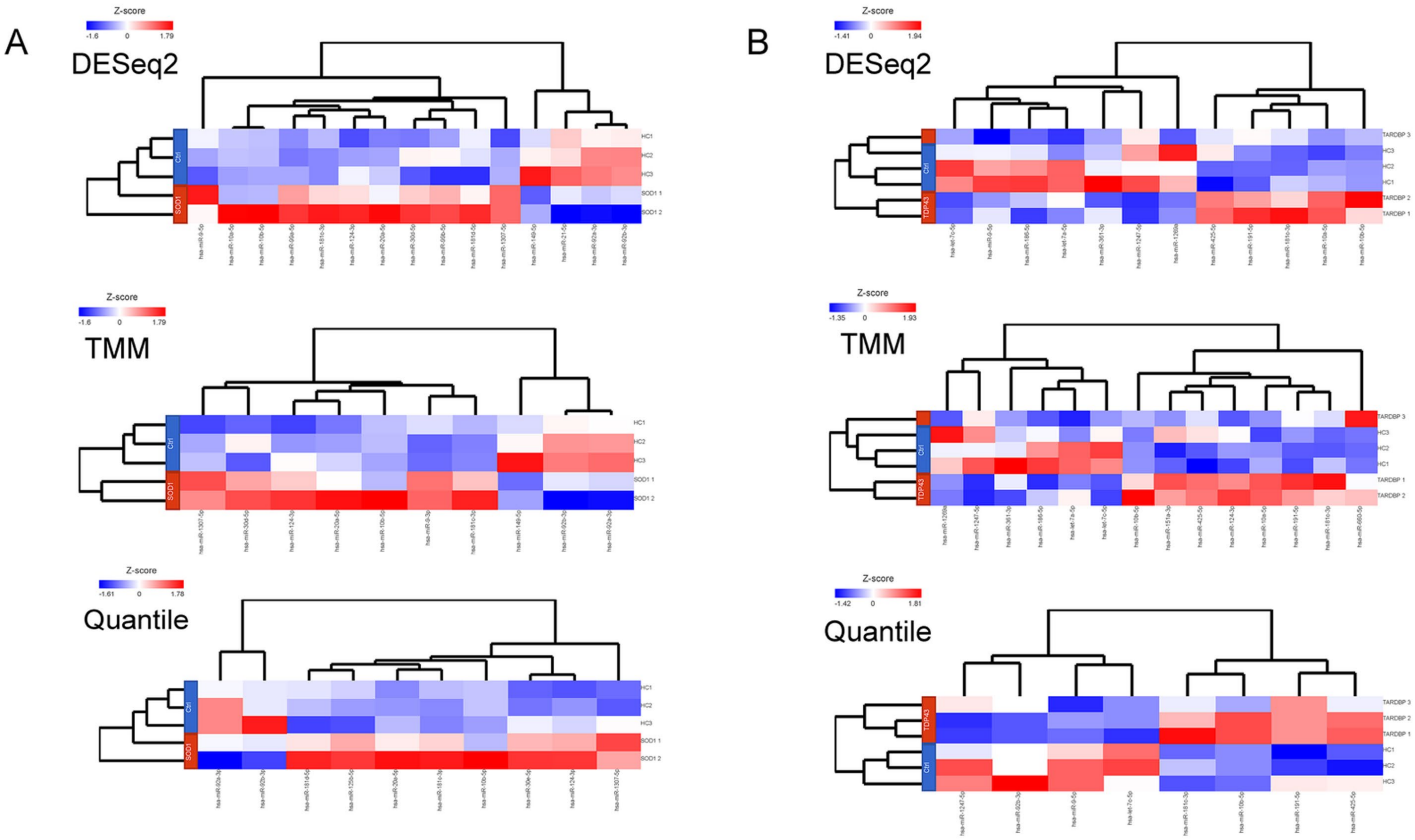
(A) Heatmaps representing the number and DE-miRNAs identified in a discovery set by each method. Expression values were normalized across *SOD1* mutant MN (*SOD1* 1, *SOD1^D90A^*; *SOD1* 2, *SOD1^R115G^*) and (B) *TARDBP* mutant MN (*TARDBP* 1 and *TARDBP* 2 (2 clones from one patient), *TARDBP^G294V^*; *TARDBP* 3, *TARDBP^S393L^*) samples (rows: ALS patients; HC, healthy controls) by Z-score, and miRNA clustering (columns) was applied. Color scale at the left of the heatmap represents the Z-score ranging from blue (low expression) to red (high expression), respectively.

When the three differential gene expression analysis methods were combined, a set of 7 (*SOD1*) and 6 (*TARDBP*) DE-miRNAs was (fold change ≥ 1.5, *p* ≤ 0.05) shared in ALS patients compared to controls (15 and 12 DE-miRNAs in DESeq2, 10 and 14 DE-miRNAs in limma-voom, 10 and 8 DE-miRNAs in quantile normalization) (Figures 1A,B), and from those miRNAs, upregulation of miR-10b-5p and miR-181c-3p was commonly found between *SOD1* and *TARDBP* mutant MNs. Figures 2A,B exhibited distinct expression patterns between *SOD1* and *TARDBP* mutant MNs versus healthy controls, respectively. Due to the robust upregulation of miR-10b-5p, we decided to focus on this miRNA for further analysis (Figures 1C,D).

### 3.2 miR-10b-5p is upregulated in neuronal and non-neuronal biomaterials of familial and sporadic ALS patients

**Figure 3:**
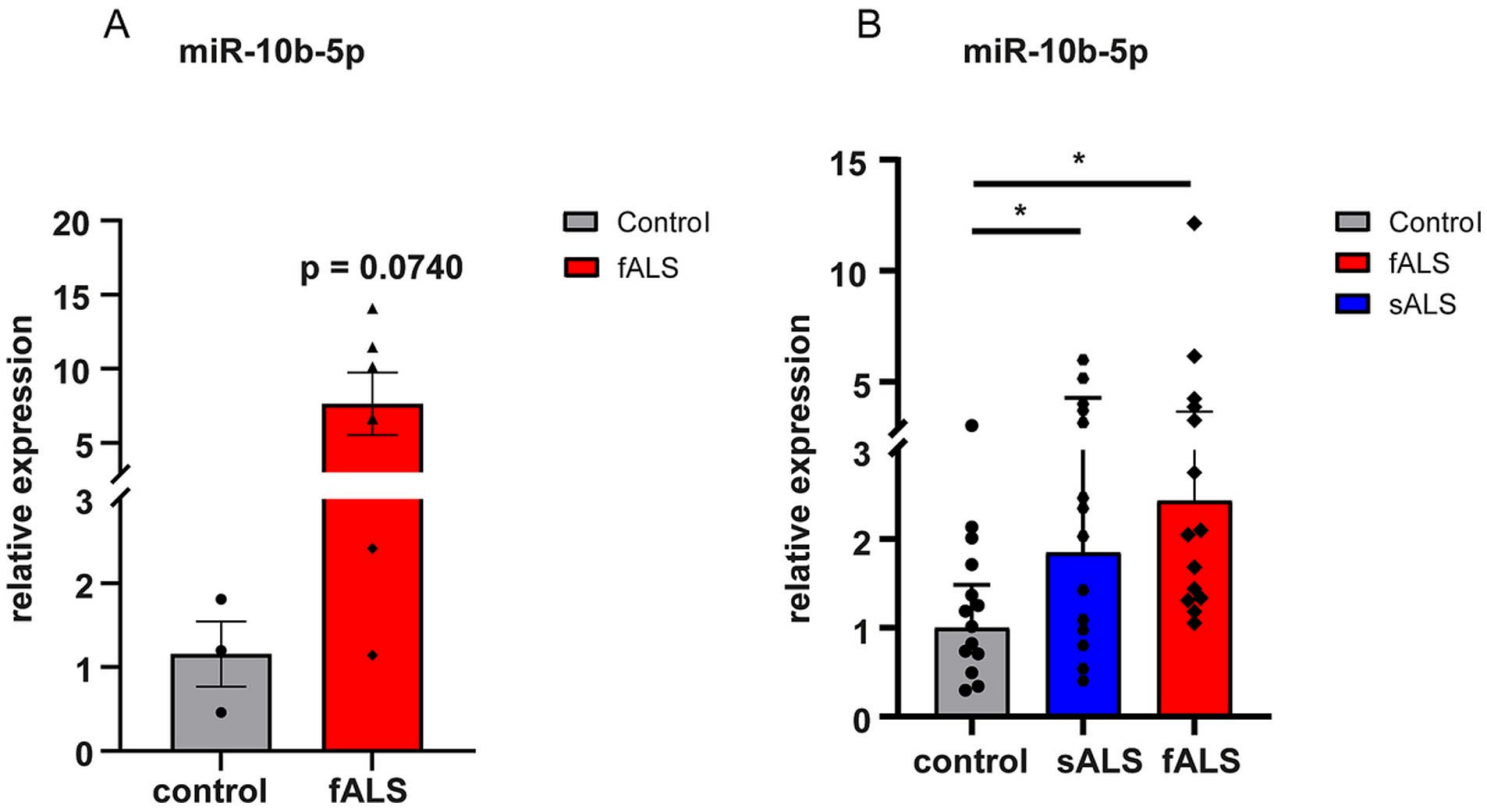
Dysregulation of miR-10b-5p by qPCR in a validation set. miRNAs were normalized to U6 snRNA. (A) Results are presented enclosing ALS postmortem subjects presenting *SOD1* or *C9orf72* mutations in a unique biological group (*n* = 6) versus healthy controls (*n* = 3). The increase of miR-10b-5p levels did not reach statistical significance but showed a strong trend (*p* = 0.0740) in fALS patients compared to healthy controls and the lack of statistical significance is rather due to the small sample number available than to indistinct results. (B) Bar plots of relative expression of miR-10b-5p detected in LCLs of healthy controls (*n* = 14), fALS (*n* = 14) and sALS (*n* = 14) patients. Genetic backgrounds of the LCLs are indicated by the name of the familial genes *SOD1*, *FUS*, *TARDBP*, and *C9orf72* carrying the mutation for LCLs or by sALS for LCLs. Compared to healthy controls miR-10b-5p levels were significantly increased in fALS mutant LCLs as well as in LCLs derived from sALS patients. Dots represent mean relative expression values of each sample [*p ≤ 0.05, **p ≤ 0.01, ***p ≤ 0.001 in a two-tailed Student’s *t*-test (Bonferroni), bars indicate ± SEM; asterisks represent *p*-values compared to the healthy controls].

Upregulation of miR-10b-5p was validated with quantitative real-time PCR using LCLs and postmortem tissue samples (brain stem) from ALS patients. Postmortem samples were available from healthy controls and fALS patients carrying mutations in *SOD1* or *C9orf72* (details see Supplementary Table S2). We had no details on postmortem delays, but there was no significant difference in the age at death or RNA integrity number (RIN) values, a measure of tissue quality, between the groups. RIN mean values were 3.46 ± 0.6754 (mean ± S.D) in ALS patients and 2.77 ± 0.6351 (mean ± S.D) in healthy controls (*p*-value = 0.1711), respectively. Similar to iPSC-derived MNs, the expression of miR-10b-5p levels were strikingly higher in postmortem central nervous system (CNS) tissue of fALS patients compared to healthy controls (Figure 3A; Supplementary Table S2). However, most likely due to the small sample size, this comparison missed statistical significance (*p* = 0.0740), but a clear trend was evident. LCLs were derived from healthy controls and fALS patients carrying causative mutations in different genes (*SOD1*, *TARDBP*, *FUS*, *C9orf72*). Details on demographics can be found in Supplementary Table S3. Interestingly, upregulation of miR-10b-5p was also detectable in non-neuronal LCLs (blood) fALS patients, but less pronounced than in iPSC-derived MNs or postmortem brain stem tissue (Figure 3B). Even more exciting, upregulation of miR-10b-5p was also detectable in LCLs of sporadic patients, suggesting that our bioinformatics approach indeed yielded in a single candidate which could not only be found in *in vitro* cell modes, but also CNS tissue and peripheral blood of patients. Moreover, it was also identified as a promising candidate for genetic and sporadic ALS patients.

### 3.3 Target gene prediction and functional enrichment/network analysis

**Figure 4:**
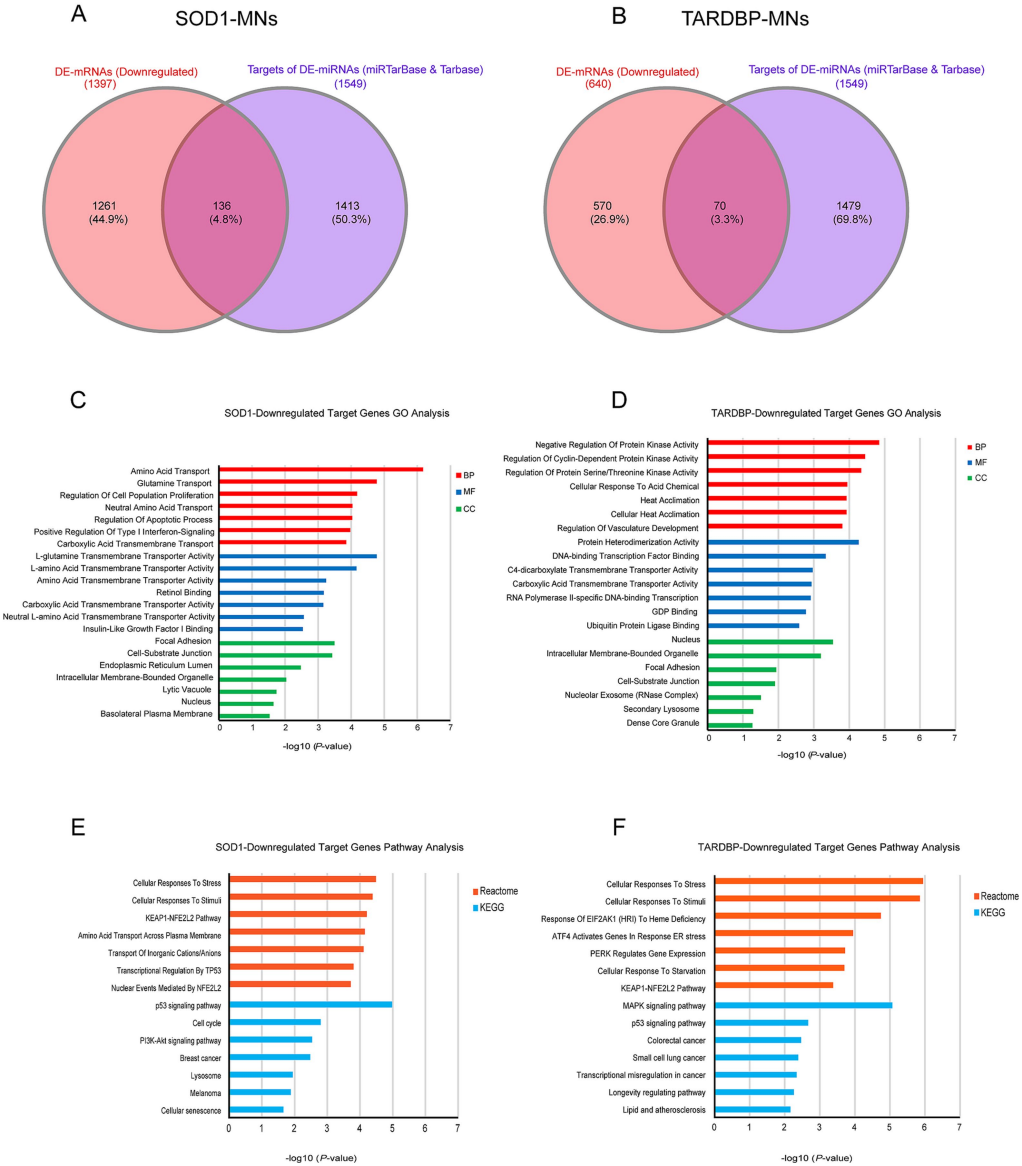
miR-10b-5p target gene analysis. **(A)** Venn diagram showing the relations between validated experimental miR-10b-5p targets and mRNAs upregulated in *SOD1* MNs and **(B)** *TARDBP* MNs, as resulting from the RNA-seq analysis. **(C)** GO enrichment analysis of miR-10b-5p in *SOD1* MNs. **(D)** GO enrichment analysis of miR-10b-5p in *TARDBP* MNs. Analysis was carried out by EnrichR illustrating significantly downregulated processes/functions (Gene Ontology; biological process, molecular function and cellular component) for the genes targeted by miR-10b-5p, respectively. Y-axis represents the GO/pathway term, and the *X*-axis represents the enrichment significance (−log_10_ (*p*-value)). **(E)** Pathway enrichment analysis of miR-10b-5p in *SOD1* MNs. **(F)** Pathway enrichment analysis of miR-10b-5p in *TARDBP* MNs. Enriched Reactome and KEGG Pathway analysis performed on the identified downregulated target genes of miR-10b-5p in *SOD1*-MNs and *TARDBP*-MNs patients (versus healthy controls) using EnrichR tools. *Y*-axis represents the pathway terms, and the X-axis represents the enrichment significance (−log_10_ (*p*-value)) *p*-value, in which the terms containing more genes tend to have a more significant *p*-value. Pathway analysis revealed the alteration of multiple pathways in *SOD1*-MNs and *TARDBP*-MNs.

**Figure 5:**
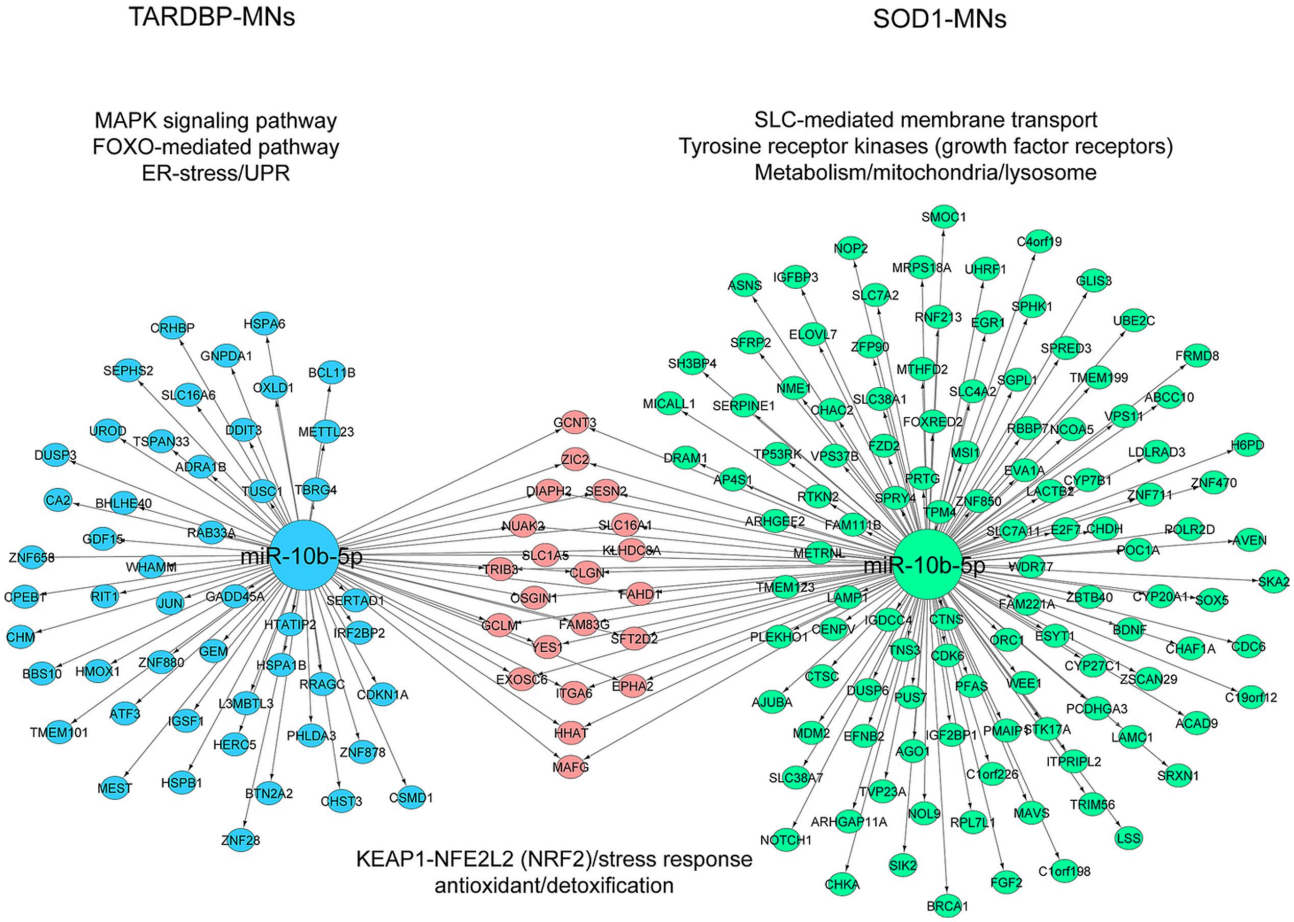
Illustration of the miRNA-target mRNA network. The resulting miRNA-target gene interactions for the common miR-10b-5p are visualized as a network with significantly (*p* ≤ 0.05) enriched shared/unique pathway terms included for each subnetwork in *SOD1* MNs and *TARDBP* MNs. miRNA and target genes are marked green and blue circles; shared genes marked pink circles. The colors of pathway terms indicate their membership in the respective networks.

To address target genes and related pathways associated with the validated miR-10b-5p, we intersected experimentally validated miR-10b-5p targets [from miRTarBase (Huang et al., 2022) and DIANA-TarBase (Karagkouni et al., 2018) databases] with our previously published mRNA expression data of the same samples (available at GEO: GSE210969) (Dash et al., 2022). Venn analysis revealed that 4.84% (136 out of 2,810) and 3.3% (70 out of 2,119) of downregulated mRNAs are putative targets of miR-10b-5p in *SOD1* and *TARDBP* mutant MNs, respectively, (Figures 4A,B). GO analysis of downregulated miR-10b-5p targets revealed a significant enrichment for GO term categories, suggesting involvement of this miRNA in cell-growth and development-related processes and functions (Figures 4C,D). Among the most enriched pathways by miR-10b-5p were mainly related to neurodegeneration, such as “p53-signaling mediated cell cycle regulation” and “metabolism or membrane transport” associated functions in *SOD1* mutant MNs, whereas functional analysis of the *TARDBP* mutant MNs showed enrichment of the “mitogen-activated protein kinase (MAPK) signaling pathway” and “FOXO-mediated pathway,” which were implicated in a wide variety of cellular processes including cell differentiation, survival and apoptosis processes (Figures 4E,F). Interestingly, target genes of miR-10b-5p shared between *SOD1* and *TARDBP* mutant MNs were enriched in pathways related to the transcription nuclear factor erythroid 2-related factor 2, and its principal negative regulator, KEAP1, Kelch-like ECH (erythroid cell-derived protein with CNC homology)-associated protein 1 (KEAP1-NFE2L2 (NRF2)), which play key roles in the regulation of antioxidant stress response against oxidative (and chemical stress) and maintain redox homeostasis. We also assessed the presence of putative functional interactions in both *SOD1* and *TARDBP* mutant MNs with a miRNA^up^-mRNA^down^ gene network showing unique/shared RNA profile signatures that may participate in downregulated gene expression observed during ALS disease (Figure 5).

## 4 Discussion

ALS is a dreadful and yet mostly untreatable neurodegenerative disease. This might be true due to the heterogeneity of the putatively underlying disease mechanisms including the lack of pathophysiology defining biomarkers. This is particularly true for sporadic cases. A global dysregulation of miRNAs in MNs has been reported in ALS patients compared to healthy controls (Dash et al., 2024; Emde et al., 2015; Taylor et al., 2016). However, whether associate miRNA expression changes simply reflect biological dysfunction and MN degeneration, or is responsible to promote or suppress specific functions of target genes relevant to ALS disease progression, remains elusive. Further, very few studies (Dash et al., 2024; De Santis et al., 2017; Rotem et al., 2017) have gained increasing interests in systematically analyzing bioinformatic or experimental biological interpretation of predicted functional targets regulated by ALS-related miRNAs in the same subjects (ALS patients versus healthy controls). Owing to advanced detection and statistical annotation methods, in this study, we expand our previous work with the overall aim to narrow down the top-regulated DE-miRNAs to the most important and generalisable pathomechanistic dysregulations in ALS as putative novel biomarkers of the diseases. (Dash et al., 2024). We performed an unbiased systematic pipeline for mRNA and miRNA profiling using a combination of three different normalizations to investigate both mRNA-miRNA expression in iPSC-derived MNs from *SOD1*- and *TARDBP*-ALS patients (versus healthy controls).

We thereby identified miRNA-10b-5p as utmost as the utmost regulated miRNA in those iPSC-derived MNs and could validate this in LCLs and postmortem tissues, respectively, both in sALS and fALS patients. We showed that the upregulation of miR-10b-5p may regulate several genes which are associated with biological pathways/processes potentially relevant to the pathomechanism of ALS. Particularly, in *SOD1* mutant MNs we found that cell growth/differentiation and p53-signaling were the most enriched pathways for the miR-10b-5p target genes, which is in line with several studies demonstrating significant involvement of p53 and related cyclin-dependent kinases (CDKs) in various cell responses to stresses, such as DNA damage, hypoxia and MN cell death both in spinal MNs of ALS patients and in ALS mouse models (Dash et al., 2024; Ranganathan and Bowser, 2010). In fact, our enrichment analysis showed a downregulation of several CDKs target genes involved in cell cycle and stress response processes in *SOD1*. That would also explain the increased expression of miR-10b-5p in mutated MNs and consequent downregulation of CDKs may contribute to prevent the re-entry in cell cycle at early disease onset, thus suggesting a protective role in the *SOD*1-ALS. Interestingly, the genes that undergo enrichment in MNs of *TARDBP* mutant associated with MAPK signaling, of which impaired function was previously described in several ALS models (Sahana and Zhang, 2021). More specifically, even if both fALS (multiple subtypes) and sALS can have different pathophysiological mechanisms, we believe that finding a common target dysregulated in both ALS forms can be useful either to understand the mechanism or to find a common therapeutic strategy. Reinforcing this idea, our functional enrichment analysis identified that miR-10b-5p might regulate a common set of dysregulated target genes involved in KEAP1-NFE2L2 (NRF2) stress response pathways in both *SOD1* and *TARDBP* mutant MNs, raising the possibility for common features in the disease mechanism of both models. Moreover, previous studies have indicated that the KEAP1-NRF2 pathway is dysregulated in ALS animal models (Bono et al., 2021; Mimoto et al., 2012), as well as in postmortem ALS motor cortex/spinal cord samples and a reduction in both *NFE2L2* mRNA and NRF2 protein has been observed in ALS patient tissues (versus healthy controls) (Sarlette et al., 2008). In addition, reduced KEAP1-NRF2 activity has been also implicated as a hallmark of the ALS-FTLD spectrum of neurodegenerative diseases (Bergström et al., 2014; Goode et al., 2016). Consistent with above findings, we hypothesize a plausible mechanism that miR-10b-5p may involve in the maintenance of neuronal survival/viability through modulating the cell response to oxidative/chemical stresses. This is especially true when individual miRNAs can target multiple genes, and conversely a single gene can be targeted by many miRNAs (Friedman et al., 2009). Moreover, considerable evidence suggested the existence of common downstream pathways, at least for some ALS-related genes such as SMN1/FUS (Yamazaki et al., 2012), TDP43/ATXN2 (Elden et al., 2010) or PFN1/TDP43 (Wu et al., 2012). It is thus tempting to hypothesize that those alterations in miR-10b-5p expression resulting from specific mutations in ubiquitously expressed genes (as *TARDBP*, *FUS*, *C9ORF72* or *SOD1*) or in sporadic cases of ALS might be represented as active participants in the convergence of different ALS target genes on common pathways. Therefore, future functional studies are indeed to confirm the relevance of the miR-10b-5p converge on shared pathways including both *SOD1* and *TARDBP* (and other ALS genes) relevant to MN neurodegeneration.

Although, however, there were many biomarker studies and/or therapeutic strategies have been identified in ALS, the exact mechanism underlying the expression of miR-10b-5p (upregulation/downregulation) remains unclear. Thus far, miR-10b-5p direct association has been investigated in only a few ALS studies (Alvia et al., 2022) (postmortem), (De Felice et al., 2018) (whole blood), (Si et al., 2018) (muscle tissue), and (Banack et al., 2020) (plasma) with inconsistent or contradictory results across studies. In postmortem prefrontal cortex, miRNAs alterations were shown to be able to distinguish Parkinson’s disease (PD) with and without dementia, and miR-10b-5p was positively associated with the age of onset (Hoss et al., 2016). Particularly, high levels of miR-10b-5p were reported in postmortem brain tissues and blood (plasma) samples of Huntington disease (HD) patients relative to controls and negatively correlated with the age of disease onset, with higher miR-10b-5p levels associating to an earlier age of HD onset (Hoss et al., 2015a; Hoss et al., 2015b). Therefore, further biochemical characterization of miR-10b-5p in the context of ALS and neurodegenerative diseases is urgent to better understand its potential role as a therapeutic biomarker for disease progression.

Importantly, miR-10b-5p involved in apoptosis, autophagy and other biological processes (Seong et al., 2021) and specific upregulation has been shown to regulate/suppress major target genes including class I homeobox (HOX) and brain-derived neurotrophic factor (BDNF) in the postmortem prefrontal cortex tissue and blood samples (versus healthy controls) and overexpression of miR-10b-5p in PC12 HTT-Q73 HD cells increased survival and cell viability, suggesting the activation of miR-10b-5p may have a neuroprotective role, presumably through post-transcriptionally regulating BDNF expression (Hoss et al., 2014; Hoss et al., 2015a; Müller, 2014). Consistent to our findings, dysregulation of miR-10b-5p has been implicated in sporadic ALS pathogenicity where it was significantly upregulated in whole blood samples (De Felice et al., 2018), and confirmed in postmortem tissues of prefrontal cortex and spinal cord specimens from ALS patients (Alvia et al., 2022). Therefore, the levels of miR-10b-5p may be probed as a diagnostic biomarker for ALS. In addition, we also identified decreased levels of BDNF, in *SOD1*-ALS MNs, which is a key growth factor required for the cell growth/neuronal survival in the brain, whereas reduced expression has been associated with neuronal dysfunction and death in several neurological disorders including ALS (Alvia et al., 2022; Lanuza et al., 2019).

Cellular crosstalk is an important phenomenon that regulates various and diverse ways of cell–cell communication under normal physiological and pathological conditions. Moreover, while our study highlights the potential of miR-10b-5p gene expression changes associated with sALS/fALS in MNs, LCLs, and postmortem tissues, studying the interactions between these cells including serum or blood-CSF fluid barrier is of utmost importance. Additional studies are required further define correlations of brain miR-10b-5p-levels and blood/CSF during life in individuals with ALS. Recent study reported the utility of select miR-181, largely expressed in neurons, biomarker in blood and miR-181 expression levels in serum have recently been associated with a greater risk of death in ALS (Magen et al., 2021). Along this line, in our MN datasets we also found that miR-181c-3p was strongly upregulated in the MNs of *SOD1*- and *TARDBP*-ALS, whereas miR-124-3p blood biomarker was specifically expressed in *SOD1*-MNs, suggesting an independent regulation/mechanism of specific miRNAs in the different compartments. The latter would also fit the hypothesis of miR-124-3p targets that may be used as a blood biomarker in ALS patients (Vaz et al., 2021). This is in consistent with the individual role of single miRNAs which can also be tissue or even cell-type dependent. In this context, future transcriptional profiling studies (both the mRNA and miRNA levels) combined with experimental follow-up in LCLs as well in postmortem tissue including a larger sample size/homogenous cohorts are warranted to confirm any significant correlations between dysregulated miRNAs/pathways (e.g., miR-10b-5p) and disease severity/duration in ALS.

Besides finding miR-10b-5p significantly upregulated in whole blood and postmortem tissue (prefrontal cortex) from ALS patients, several lines of evidence reported that miR-10b-5p was also overexpressed in a wide variety of diseases such as diabetes (Singh et al., 2021), breast cancer (Bozkurt et al., 2021) and brain cancer/glioblastoma (Teplyuk et al., 2016) associated with high-grade glioma and poor prognosis (Ji et al., 2015). Importantly, miR-10b-5p is downregulated (or not expressed) in normal brain cells while being upregulated and specifically expressed in cancerous brain tissues of glioma patients/glioma cell lines and that ablation of miR-10b is lethal in glioma cells (Gabriely et al., 2011; Li et al., 2019). This underpins a putative cell protective role of miR-10b-5p. In line with this information, miRNA expression profiling can also be used to correlate disease stage/clinical variables in various diseases.

There are also some limitations in our study. Even though we validated the upregulation of miR-10b-5p in LCLs and postmortem tissues from sALS and fALS patients, a broader validation in iPSC-derived MNs is needed including also other Mendelian gene mutations of fALS. Further, although the current data showed that there was an inverse correlation between expression of the miR-10b-5p and gene targets, it is thus important to validate this finding using, e.g., dual-luciferase reporter assay, as well as rescue experiments. Moreover, future studies of additional brain samples using broader populations of brain and spinal cord might strengthen the finding of our results and explore the potentiality for miR-10b-5p-mediated gene regulation of different pathways and key targets during cell survival and development in ALS neurodegeneration.

## 5 Conclusion

In conclusion, our bioinformatics pipeline combining three different ways of normalization indeed was helpful to identify few top hit DE-miRNAs. The validation in brain tissue of fALS and blood LCLs of fALS and sALS patients indicate that the upregulation of miR-10b-5p might be a general feature of ALS pathophysiology, but that it’s unclear if it’s a protective adaptation or a pathogenic change contributing to the diseases. Moreover, that perturbed miRNA expression could be a common molecular ground of multiple subtypes of ALS to understand the pathogenesis. Therefore, further functional studies will be necessary to unravel the pathomechanisms underpinning the involvement of miR-10b-5p in *SOD1* and *TARDBP*-ALS neurodegeneration, but worth doing because miR-10b-5p may turn out as a potential therapeutic target, and normalization of expression may have beneficial effects in several pathways including KEAP1-NRF2 stress response associated with neurodegeneration.

## Data Availability

The datasets presented in this study can be found in online repositories. The names of the repository/repositories and accession number(s) can be found in the article/Supplementary material.
