# Supplementary material for: Upregulated miR-10b-5p as a potential miRNA signature in amyotrophic lateral sclerosis patients
Source: Front Cell Neurosci. 2024 Nov 7;18:1457704. doi: 10.3389/fncel.2024.1457704 (PMC11586771; doi:10.3389/fncel.2024.1457704)
Supplement: Supplementary file 4 [file Data_Sheet_1.docx]

Supplementary Material

# Supplementary Figures and Tables

## Supplementary Figures


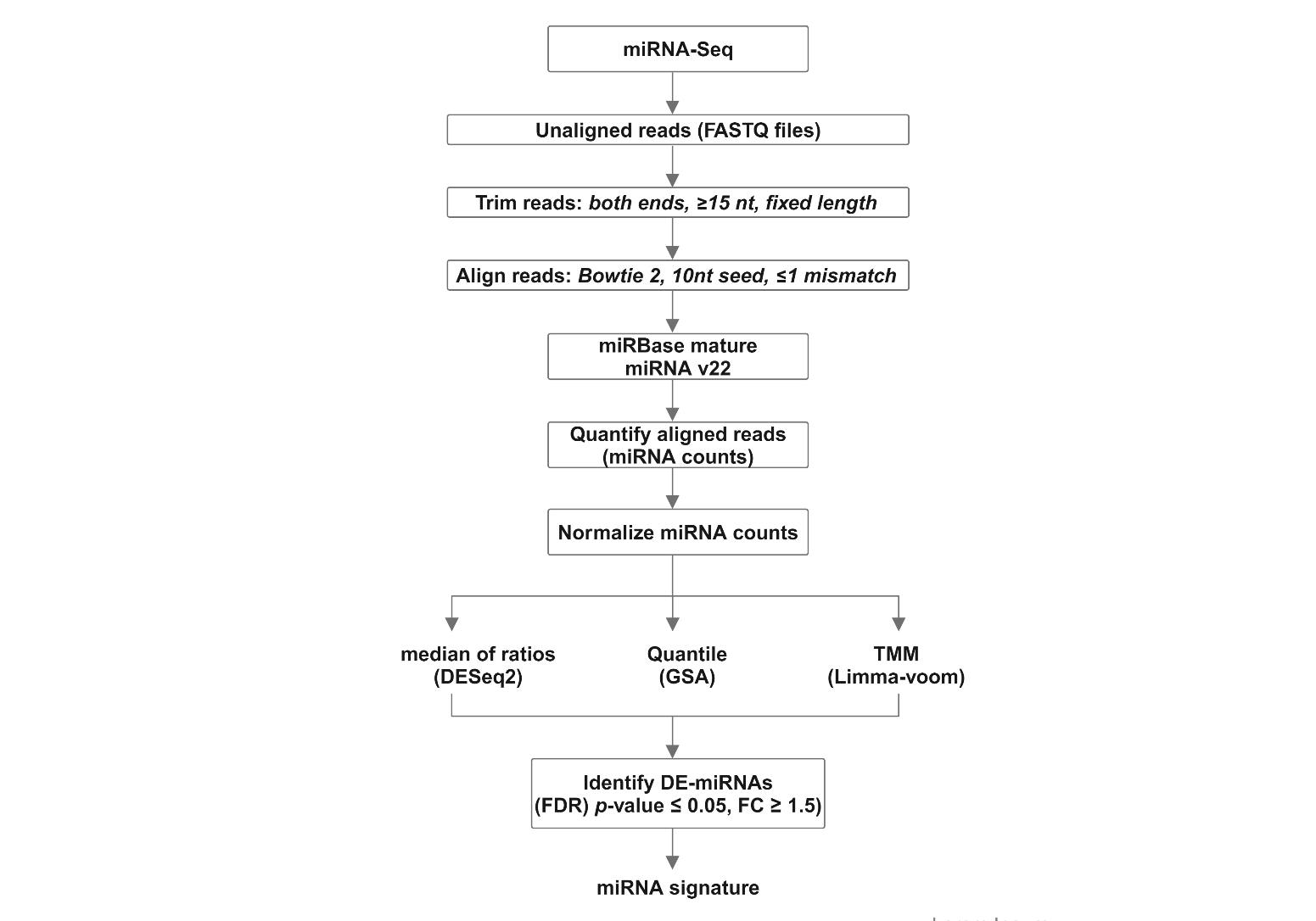


**Supplementary Figure 1.** Data processing workflow.

## Supplementary Tables

| **Group**  **(n)** | **Sex** | **Age at biopsy (years)** | **mutation** | **Age at disease onset** | **Clinical phenotype** | **Disease duration (months)** |
| --- | --- | --- | --- | --- | --- | --- |
| Controls  (3) |  |  |  |  |  |  |
|  | Female | 53 | - | - | - | - |
|  | Female | 45 | - | - | - | - |
|  | Male | 60 | - | - | - | - |
| SOD1  (n=2) | Female | 46 | D90A | 41 | Spinal | 204 |
|  | Male | 59 | R115G | n.d | Spinal | n.d |
| TARDBP  (n=3) | Female | 85 | S393L | 85 | Bulbar | 48 |
|  | Male | 46 | G294V | 37 | Spinal | >120 (alive) |

n.d.; no data

**Supplementary Table 1.** Patient/proband characteristics of iPSC lines.

|  | mutation | gender | age [years] |
| --- | --- | --- | --- |
| controls (n = 3) | / | male | 67 |
|  | / | male | 63 |
|  | / | male | 74 |
| patients (n = 6) | SOD1 D90A | female | 63 |
|  | SOD1 D90A | male | 75 |
|  | SOD1 G127X | male | 53 |
|  | SOD1 D90A | male | 53 |
|  | C9orf72 | male | 65 |
|  | C9orf72 | male | 61 |

**Supplementary Table 2.** Natively frozen brain stem tissue was derived from healthy controls and fALS patients autopsied at Umeå Univeristy.

|  | age at sampling [years] | sex | age at onset [years] | site of onset | disease duration [months] | Gene | variant |
| --- | --- | --- | --- | --- | --- | --- | --- |
| controls | 49 | m | - | - | - | - | - |
|  | 40 | m | - | - | - | - | - |
|  | 37 | f | - | - | - | - | - |
|  | 73 | f | - | - | - | - | - |
|  | 70 | f | - | - | - | - | - |
|  | 46 | f | - | - | - | - | - |
|  | 44 | f | - | - | - | - | - |
|  | 61 | f | - | - | - | - | - |
|  | 60 | m | - | - | - | - | - |
|  | 61 | f | - | - | - | - | - |
|  | 74 | m | - | - | - | - | - |
|  | 77 | f | - | - | - | - | - |
|  | 75 | f | - | - | - | - | - |
|  | 46 | m | - | - | - | - | - |
| sALS | 52 | m | 50 | spinal | 74 | - | - |
|  | 63 | f | 60 | bulbar | 39 | - | - |
|  | 69 | m | 67 | bulbar | 25 | - | - |
|  | 55 | f | 53 | bulbar | 106 | - | - |
|  | 69 | f | 67 | spinal | 25 | - | - |
|  | 54 | m | 53 | bulbar | unknown | - | - |
|  | 60 | m | 59 | spinal | 48 | - | - |
|  | 63 | m | 60 | spinal | 29 | - | - |
|  | 49 | f | 47 | bulbar | 31 | - | - |
|  | 63 | f | 60 | spinal | 49 | - | - |
|  | 58 | m | 55 | bulbar | 38 | - | - |
|  | 38 | f | 37 | spinal | 32 | - | - |
|  | 67 | f | 66 | bulbar | 21 | - | - |
|  | 52 | f | 51 | spinal | unknown | - | - |
| fALS | 68 | m | 60 | spinal | 201 | SOD1 | D90A |
|  | 53 | f | 52 | bulbar | 47 | C9orf72 | HRE |
|  | 39 | f | 33 | spinal | 283 (still alive) | SOD1 | I104F |
|  | 41 | m | 41 | bulbar | 15 | FUS | R514G |
|  | 74 | m | 72 | spinal | unknown | SOD1 | D90A |
|  | 40 | f | 39 | spinal | 221 | TARDBP | N352S |
|  | 70 | f | 67 | spinal | 37 | FUS | K510R |
|  | 40 | f | 28 | thoracic | 251 | SOD1 | E100K |
|  | 60 | f | 55 | spinal | 98 | TARDBP | N352S |
|  | 65 | f | 63 | spinal | unknown | SOD1 | G72S |
|  | 56 | m | 48 | spinal | 98 | C9orf72 | HRE |
|  | 71 | m | 70 | spinal | 9 | SOD1 | I113T |
|  | 46 | f | 44 | generalized fasciculations, no paresis | unknown | SOD1 | H43R |
|  | 77 | m | 73 | spinal | 59 | SOD1 | V87A |

**Supplementary Table 3.** Details on patients and probands from whom we lymphoblasoid cell lines (LCLs) were derived.
